# Increased Attraction and Stability of *Beauveria bassiana*-Formulated Microgranules for *Aedes aegypti* Biocontrol

**DOI:** 10.3390/jof8080828

**Published:** 2022-08-08

**Authors:** Norma Zamora-Avilés, Alonso A. Orozco-Flores, Ricardo Gomez-Flores, Maribel Domínguez-Gámez, Mario A. Rodríguez-Pérez, Patricia Tamez-Guerra

**Affiliations:** 1 Facultad de Ciencias Biológicas (FCB), Universidad Autónoma de Nuevo León (UANL), Av. Pedro de Alba S/N, Cd. Universitaria, San Nicolás de los Garza 66455, Nuevo León, Mexico; 2 Centro de Biotecnología Genómica, Instituto Politécnico Nacional, Reynosa 88710, Tamaulipas, Mexico

**Keywords:** propagules stabilizers, mosquito control, conidia viability

## Abstract

*Aedes aegypti* (Linn.) incidence has increased in recent years, causing human viral diseases such as dengue, which are often fatal. *Beauveria bassiana* (Bals., Vuillemin) efficacy for *Ae. aegypti* biological control has been evidenced but it relies on host susceptibility and strain virulence. We hypothesized that *B. bassiana* conidia microgranular formulations (MGF) with the additives acetone, lactic acid, and sugar increase *Ae. aegypti* adult exposure, thus improving their biocontrol effectiveness. *Beauveria bassiana* strain four (BBPTG4) conidia stability was assessed after 0 d, 5 d, and 30 d storage at 25 °C ± 2 °C with additives or in MGF after 91 d of storage at 25 °C ± 2 °C or 4 °C ± 1 °C, whereas mortality was evaluated after adult exposure to MGF + conidia, using home-made traps. Additives did not show toxicity to conidia. In addition, we observed that sugar in MGF increased *Ae. aegypti* adults’ attraction and their viability resulted in a 3-fold reduction after 5 d and 1- to 4-fold decrease after 30 d of storage, and formulations were less attractive (*p* < 0.05). Conidia stability was higher on MGF regardless of the storage temperature, losing up to 2.5-fold viability after 91 d. In conclusion, BBPTG4 infected and killed *Ae. aegypti*, whereas MGF attracting adults resulted in 42.2% mortality, increasing fungus auto dissemination potential among infected surviving adults. It is necessary to further evaluate MGF against *Ae. aegypti* in the field.

## 1. Introduction

*Aedes aegypti* populations and prevalence have recently increased. This mosquito is the vector agent of several arboviral diseases, including dengue, which is currently present in 128 countries [1,2]. In addition, zika virus has been reported in the Americas and the Pacific, causing outbreaks, and threatening public health due to its association with neurological complications [3]. It has been estimated that half of the world population has been exposed to diseases transmitted by this mosquito vector [4].

*Ae. aegypti* control includes integrated vector management, vector surveillance based on health information systems, and emergency preparedness. Unfortunately, evaluating these control components in Latin America and the Caribbean has proven to be unsuccessful, because they have not been properly addressed [5]. Insects’ development of resistance by frequent exposure to chemical insecticides reduces arbovirus control [6], which leads to an increasing interest in other management tools for this dengue vector, including entomopathogenic fungi (EPF) application [7]. Selected *Beauveria bassiana* (Bals.) Vuillemin strains infect and kill adult mosquitos by direct exposure or by horizontal dissemination via copula [8].

After application, fungi-based bioinsecticides are threatened by environmental conditions, such as high temperature and direct solar radiation. Low humidity is also a limiting factor for fungal infection and mycelium development on dead insects [9]. Nevertheless, conidia viability increases at low relative humidity (RH) [10]. Fungal propagules are protected after being formulated, because ingredients reduce the effects of environmental conditions on fungus virulence, improving their stability and residual activity after application [11,12,13].

Infection process begins with direct contact of fungus propagules to the insect’s cuticle, which may reduce fungus activity and biological control efficacy. To improve their contact rate and effectiveness, an additive is included in fungal formulations. Granular formulations have been used to encapsulate entomopathogens since the late 1970s, whose materials are selected based on their potential to form matrices (by retrogradation), when combined with water, showing cost-effective results, particularly using *B. bassiana* GHA and *Metarhizium robertsii* strains [14].

A wide variety of water-soluble ingredients are available to prepare granules and microgranules [15,16]. The selected material is considered the granule matrix, to which the bioactive agent and other desired additives (sunscreens, phagostimulants, adherents, and even fungal growth inhibitors) are incorporated [17,18]. For instance, the commercial product *Bacillus thuringiensis israelensis* strain MH14 (Bioflash^®^) is granular and only recommended for mosquito larvae biocontrol [19]. Furthermore, the use of microorganisms such as the microalgae *Spirulina* may provide stability to conidia due to their fat content (11% of lipids) [20] and increase the attraction upon the activation of the bioformulation with the addition of water, by generating a greater amount of CO_2_ through its photosynthetic activity [21].

However, the smell of human or animal hosts is the major known attraction factor to mosquitoes [22]. It has been reported that CO_2_ and acetone attract *Ae. aegypti* because they are the main respiration components [23]. In human skin, the additive L-lactic acid is the glycolysis final product during anaerobic metabolism. Other semiochemicals, such as amino acids, octanol, and carboxylic acids, have been shown to be effective additives for *Ae. aegypti* adults [24,25].

Therefore, it is necessary to develop formulations against mosquitos that improve the stabilization of their active agents, increase shelf-life, provide probiotic stability against environmental exposure, and improve their efficacy. Formulations prepared with *B. thuringiensis* (Bt) as an active ingredient and k-carrageenan hydrogels as a matrix have been reported to provide stability to Bt, achieving 100% *Ae. aegypti* larval mortality after 11 wk of exposure [26]. Furthermore, several studies have shown that *Metharrizium anisoplae* (Metsch.) Sorokin [11] and *B. bassiana,* tested as active ingredients, were effective to control *Ae. aegypti* adults [27,28,29,30]. However, an effective formulation with the required characteristics for EPF to remain viable and be effective after application has not yet been demonstrated.

The aim of the present study was to evaluate additives and a microgranular formulation in combination with *B. bassiana* conidia to increase stability and improve *Ae. aegypti* adults’ attraction and biocontrol.

## 2. Materials and Methods

Reagents were obtained from Sigma-Aldrich *Química,* *S.A. de C.V.* (Toluca de Lerdo, México), unless otherwise specified.

### 2.1. Mosquito Source and Rearing Conditions

*Ae. aegypti* strain was provided by the Laboratorio de Entomología of Facultad de Ciencias Biológicas at Universidad Autónoma de Nuevo León, México. *Ae. aegypti* colony was kept inside of an insect breeding room at 25 °C ± 2 °C and 80% RH, following the protocol described in the Guide for the Installation and Maintenance of *Aedes aegypti* Linn. (Diptera: Culicidae) insectary (http://www.cenaprece.salud.gob.mx/programas/interior/vectores/descargas/pdf/GuiaInstalacionMantenimientoInsectario.pdf) (website accessed on 5 June 2022) from the Ministry of Health of Mexico. Adults were kept in a 38.1 cm width × 60.0 cm height pop-up butterfly cage (Carolina Biological Supply Company, Burlington, NC, USA), placed inside an insect rearing room at 25 °C ± 2 °C, 60% ± 10% RH, and 14 h light: 10 h darkness photoperiod. Mosquitoes were fed on 5% sugar solution-soaked cotton bolls in a 20 mL plastic cup, placed near a cage corner. Sugar solution was replenished daily using a 3 mL syringe, whereas females were also blood-fed by a human arm, following the Ministry of Health of México’s protocol. For oviposition, 2 L plastic cylindrical containers with 700 mL of tap water and 0.5 g of fish flakes (Wardley^®^, Grupo Acuático Lomas, S.A. de C.V., Cuajimalpa, México) were placed inside the adult cage. After emerged larvae was observed, the container top was covered with muslin mesh and the oviposition container was replaced for a new one. Larvae from neonate to fifth instar were fed with fish flakes. Pupae were transferred to cages for adult emergence and this cycle was repeated. The emerged 5- to 8-day-old *Ae. aegypti* adults were changed to a different release cage for bioassays. About 20 males and 20 females were kept untested for the maintenance of the mosquito colony under the rearing conditions described above [31].

### 2.2. B. bassiana Culture and Mass Production

*Beauveria bassiana* strain four (BBPTG4) (Genbank: KC759730), originally isolated from cockroaches and tested against *Epilachna varivestis* Mulsant (Coleoptera: Coccinellidae) larvae [32], was maintained in the Colección de Hongos Entomopatógenos of the Unidad de Formulación de Biológicos at Universidad Autónoma de Nuevo León, México. For conidia re-activation, BBPTG4 strain was grown on potato dextrose agar (PDA) (BD Difco, Ciudad de México, México) in Petri dishes (5 cm diameter × 1 cm depth) (Med Lab S.A. de C.V., Estado de México, México) from a monosporic stock. Fungus inoculation was made with 100 µL of conidial suspension. Inoculated Petri dishes were then incubated at 25 °C for 6 d to 8 d in darkness, until sporulation. Produced conidia were removed by adding 1 mL of 0.5% INEX-A^®^ (Cosmocel, Monterrey, N. L., México) as a dispersant to obtain a stock solution. We measured conidia viability from the stock suspension in all experiments. For this test, 20 µL of the suspension was set for germination counting on potato dextrose broth (PDB) (BD Difco) and incubated at 25 °C ± 2 °C. Conidia were considered viable by counting 100 conidia three times to determine germination percentage [33]. Next, the tested amount was adjusted to reach an initial concentration of 1 × 10^8^ viable conidia/mL. Once fungus viability was evidenced, 200 µL of the conidia stock was inoculated into an Erlenmeyer flask with 200 mL of PDB and incubated at 25 °C ± 2 °C in an automatic rotary shaker at 120 rpm (Orbit 1900, Labnet, Ciudad de México, México) for 5 d, until blastopore structures were detected and adjusted to 1 × 10^8^ blastospores/mL, using a Neubauer chamber under a phase-contrast microscope at 40×. This suspension was used to inoculate rice for semi-massive production by solid fermentation. 

Solid fermentation for semi-massive BBPTG4 conidia production as an active ingredient (AI) was performed as reported elsewhere [34]. In brief, 100 g of pre-moistened sterile parboiled rice grains, used as solid substrate, was placed in 800 mL glass bottles, containing 30 mL of hydration sterile solution (0.97 g/L KH_2_PO_4_, 410 µL/L of H_2_SO_4_, and 0.31 g/L yeast extract). Solid fermentation was performed by inoculating 100 g of hydrated rice grains with 1 × 10^8^ viable blastospores/mL and incubating at 25 °C ± 2 °C for 8 d to 14 d, in darkness. During incubation, rice solid culture in bottles was mixed daily with a spatula for aeration, under sterile conditions.

Rice-cultured conidia were harvested using a standard testing No. 40 sieve (426 µm opening size). Produced conidia were quantified by taking 15 mg, suspended in 0.5% INEX-A^®^ (emulsifier agent), counted, and stored at 4 °C to prepare granular formulations.

### 2.3. Formulations Production

#### 2.3.1. Viability of Conidia in Combination with Additives

Bioassays were performed before the preparation of formulations and the potential negative effect of *Ae. aegypti* additives on BBPTG4 conidia viability was immediately determined after mixing with additives (time zero) and storage at 25 °C ± 2 °C, as described above. Conidial suspensions and negative control (without additives) were prepared in 0.5% INEX-A^®^ solution. Conidial preparations were adjusted to 4.3 × 10^8^ viable conidia/mL and exposed to *Ae. aegypti* additives. Additives were prepared using 0.5% INEX-A^®^ solution as follows: 2 µg/mL (LA2), 4 µg/mL (LA4), and 8 µg/mL (LA9) lactic acid; 1% (Ac1), 5% (AC2), and 10% (AC3) (*v*/*v*) acetone; and 1% (Su1), 5% (Su2), and 10% (Su3) (*w*/*v*) sugar. BBPTG4 conidia plus additives and two controls (conidia without additives) were evaluated at 0 d, 5 d, and 30 d of storage. We prepared 9 replicate determinations for each additive concentration, using as a control, 18 replicate determinations of conidia without additives. 

To evaluate germination tube development percentage, conidia viability was determined on PDA medium in a Petri dish plate by colony forming units (CFU) count, selecting conidia dilutions (4.3 × 10^5^ conidia/mL), based on viability percentage results. CFU number was multiplied by the dilution factor to obtain the viable conidia value. We analyzed CFU means of recorded data among treatments for each time kept at 25 °C ± 2 °C for 6 d, by one-way ANOVA (*p* < 0.05) and the honestly significant difference (HSD) Tukey test for post hoc multiple means comparison. All tests were performed using the SPSS version 21.0 [35].

#### 2.3.2. Conidia Viability in Microgranular Formulations

*B. bassiana*-based microgranular formulations (MGFs) were prepared as previously reported [34,36]. In brief, a mixture containing 7.5 g of nixtamalized processed corn flour, 7.5 g of cornstarch, 0.075 g of sugar, and 5.4 mL of soybean oil constituted the dried granules total weight used as the matrix. Ingredients were mixed and homogenized by adding 10 mL of purified drinking water and MGFs without conidia (untreated) to be used as the negative control. Microgranules were obtained using a fine sieve N° 40 to pass through the dough. The estimated granule size was 0.1 mm.

For comparison, conidia viability of BBPTG4 mixed on MGF after storage was determined as described above, including three replicate determinations by formulation, storage time, and temperature. BBPTG4 was tested as an active ingredient at 1.0 × 10^8^ viable conidia/g of MGF and viability was determined at 4 d, 11 d, 18 d, 25 d, 32 d, 61 d, 77 d, and 91 d of storage at 25 °C ± 2 °C or 4 °C ± 1 °C. For the conidia viability test, formulations were mixed in distilled water with INEX-A^®^ at 0.05% vol/vol, resulting in 1.5 × 10^7^ conidia/mL. The criterion of germination was that the germ tube diameter was twice that of the spore [34]. Since ingredients used to prepare MGFs were not previously sterilized, PDA medium was supplemented with a sterile solution (by membrane filtration) containing 2 mg/L each of tetracycline, streptomycin, and penicillin (Sigma-Aldrich, St. Louis, MO, USA).

#### 2.3.3. Additives Effectiveness in MGF

##### 2.3.3.1. Attractiveness Test of MGF in *Ae. aegypti* Adults

Since additive combinations, such as lactic acid + acetone, showed to be effective for the attraction of *Ae. aegypti* and *Anopheles* sp., additives were evaluated using them alone [24] or the combination of lactic acid + acetone, which were also added to MGFs. Treatments included (a) MGF (sugar), (b) MGF + acetone at 1% (*w*/*v*) of MGF, (c) MGF + 100 µL lactic acid at 85% per gram of MGF, and (d) MGF (sugar) + 100 µL lactic acid at 85% + acetone at 1% (*w*/*v*) of MGF.

For attractiveness evaluation [37], triplicates of 30 *Ae. aegypti* adults were used [38]. For this, adults were transferred to a cage similar to that used for rearing but inside the cage we placed a conventional home mosquito trap (conventional CO_2_ bioproducing system) and the plastic cup with cotton soaked in 5% sugar solution for feeding. The conventional home trap consisted of an empty 2 L plastic soda bottle, cutting one third of the top area to achieve a funnel-shaped container, which was placed upside down on the top side of the cut bottle. The bottom of each trap was filled with 700 mL of tap water and the external surface was covered with a black paint color.

The bottom of the upside-down funnel-shaped soda bottle mosquito trap was covered with muslin and placed in a container filled with 700 mL of tap water, after which 6 g of each treatment with microgranules were added, instead of the sugar and yeast extract, to test the attractiveness by using the mosquito trap system (BG CO_2_ Mosquito Trap|Nixalite accessed on 30 June 2022).

One trap/treatment was placed inside the cage containing *Ae. aegypti*, allowing adults to reach the treatment by the narrow funnel area but avoiding the mosquitoes to reach the trap solution or to directly feed on the cotton soaked with 5% sugar solution. After 48 h, a muslin-mesh net was placed on the top of the funnel area of the trap and trap-attracted mosquitoes were immobilized by placing the whole trap inside of a refrigerator for 10 min. Next, adults were taken from the trap by removing the muslin-mesh net placed initially at the bottom area of the funnel from the trap top.

##### 2.3.3.2. *Ae. aegypti* Trap Mortality by MGF with *B. bassiana* Conidia Active Ingredient

We selected the MGF + additive treatment showing the highest adult attraction to be used with *B. bassiana* conidia as the active ingredient (AI). For this, conidia were added to MGF to reach a final concentration of 1.0 × 10^8^ conidia/g, using a control without AI. Adult attractiveness evaluation was performed as described above. The mosquitoes that entered the traps and were in contact with the MGF were collected in a second cage to record mortality. For this, alive mosquitoes were individually collected from the funnel mesh as previously described but using sterile conditions. Adults were separately placed inside of a 1 L plastic container with a cup with 5% sugar solution-soaked cotton to ensure mosquito feeding and survival, using the same incubation conditions as those for the colony. Sugar solution was added, if necessary, as explained above.

For mortality evaluation, *Ae. aegypti* adult’s survival was determined every third day for each treatment and bioassay in triplicate for up to 12 d. Dead mosquitoes were counted to register mortality percentage and placed inside of a humid chamber to visualize aerial mycelium development [39,40], using a stereoscope to confirm the presence of Bb mycelium. *Ae. aegypti* adult’s attraction and mortality percentage means were analyzed by the Student *t* test (*p* < 0.05) for independent samples. Conidia viability in combination with MGF was evaluated every 15 d for three months after storage at 25 °C ± 2 °C or 4 °C ± 1 °C, testing the remaining *B. bassiana* conidia germination percentage as mentioned above.

### 2.4. Effect of Spirulina *sp.* as Ae. aegypti Attractant or Conidia Stabilizer

#### 2.4.1. Attraction of *Ae. aegypti* Adults by *Spirulina* sp. in MGFs

Since *Spirulina* algae provides stability to conidia due to their fat content [20] and increases attraction by producing high amounts of CO_2_ [21], to improve *Ae. aegypti* adult’s attraction to MGFs, dried *Spirulina* sp. was added to the formulation dough, testing ingredients at the same amounts as detailed above (Section 2.3.2), with *Spirulina* sp. (Vidanat Alga *Spirulina*; Super Mayoreo Naturista S.A. de C.V., Ciudad de México, México) at 3.8% *w*/*w*, coding the treatment as MGFs + Sp.

We used MGF or MGF + Sp treatments for *Ae. aegypti* adult attraction and mortality evaluation inside a cage (Carolina Biological Supply Company) and inside a conventional funnel-shaped trap, releasing 15 mosquitoes inside each trap for each treatment. The mosquito number inside each trap + treatment was counted after 48 h [41].

##### 2.4.1.1. Evaluation of Attractiveness *Spirulina* sp. in Granular Formulation

We used the following treatments to evaluate attractiveness: (a) dry MGFs (the best treatment of Section 2.4.1) + 3.8% *Spirulina* (MGFs + Sp dry) and (b) humid MGFs + 3.8% *Spirulina* (MGFs + Sp hum). Triplicates of 30 *Ae. aegypti* adults were used. Formulations were dried for 17 h and treatment *b* was moistened with 6 mL of sterile purified water. *Ae. aegypti* adult attractiveness assessment was performed as described in Section 2.3.3.1. The attraction percentage was determined at 48 h by counting mosquitoes inside the traps. Percentage means of *Ae. aegypti* adult attraction were analyzed by the Tukey test for unequal groups *(p* < 0.05).

#### 2.4.2. *B. bassiana* Conidia Viability on a Granular Formulation with *Spirulina* sp.

For this experiment, we used the formulation that showed the highest *Ae. aegypti* attraction in Section 2.4.1.1. The formulation consisting of *B. bassiana*-based MGFs + Sp showed the highest *Ae. aegypti* attraction. We used 2.6 × 10^8^ conidia/g for treatments with an initial viability of 70% of the stock suspension and prepared replicate determinations for the storage temperatures 25 °C ± 2 °C and 4 °C ± 1 °C. Similarly, for shelf-life determination, 50 mg of each replicate were suspended in 0.05% INEX-A, after which 1/10 dilutions were made. We selected 4.3 × 10^5^ conidia/mL to measure spore germination, based on viability percentage results. Two millimeters thick agar sections were placed on a series of glass slides. One drop of the conidia suspension was then placed on each agar disc and slides incubated at 25 °C ± 2 °C in darkness, after which percent germination was determined after 17 h, counting viable and non-viable conidia.

Evaluations were determined at 0 d, 15 d, 30 d, and 45 d at experimental storage temperatures. Percentages means of recorded data for each treatment throughout the storage periods were analyzed by one-way ANOVA and the honestly significant difference (HSD) Tukey test (*p* < 0.05) for post hoc multiple means comparison. Germination percentage between treatments in each period was analyzed by the Student *t* test (*p* < 0.05) for independent samples [39].

### 2.5. Evaluation of Granular and Solid Formulations

#### 2.5.1. Conidia Viability on MGFs with *Spirulina* sp. and Solid Formulation (SF) with Coco Fiber

To improve MGFs + Sp formulation, we performed treatments containing flour and water as mentioned above (Section 2.3.2, developing solid formulations by adding vegetable fat or ground coconut fiber (Table 1).

MGFsSp, MGFs, SFSp, and SF treatments and controls (conidia suspended in 0.05% INEX-A^®^ or 0.05% INEX-A^®^ alone) were prepared under sterility, vacuum packed in triplicate, and stored at room and cold temperatures (Table 2). In addition, *B. bassiana* conidia shelf life was evaluated at 0 h, 24 h, 15 d, 30 d, and 120 d after preparation and viability was measured as explained above. Data were analyzed by the one-way ANOVA (*p* < 0.05) and HSD Tukey for post hoc multiple means comparison tests.

#### 2.5.2. *Aedes aegypti* Adult Infection by Microgranular Formulations and Solid Formulations

Treatments were the same used for *B. bassiana* conidia shelf life, involving MGF + sugar + *Spirulina*, MGF + sugar, solid formulation + sugar + *Spirulina*, and solid formulation + sugar that were stored at room temperature (Table 2). *Ae. aegypti* adult biocontrol efficacy was evaluated in triplicate, using 10 adults exposed for 48 h to each treatment. Treatments were replaced by cotton soaked in water solution with 5% sugar. Adult survival data were collected daily for up to 16 d. For mosquito handling, adults were immobilized for 5 min at 4 °C. The infection traps consisted of 1 L bottles and the container lid was covered with a fine mesh, where 5 g of each formulation and cotton with sugar was placed. Dead mosquitoes were then processed as explained in Section 2.3.3.2 [40].

## 3. Results

### 3.1. B. bassiana Conidial Viability after Exposure to Additives

At time zero, we observed 90% to 100% conidia viability. Nevertheless, with all tested attractants, conidial viability experienced a 1000-fold decrease within 5 d to 30 d. Lactic acid treatments induced 3- to 4-fold viability decrease, whereas 1% and 5% acetone caused 1-fold viability reduction (5.7 × 10^5^ viable conidia), compared with the resulting viability after 5 d of storage. After 30 d of storage, conidia viability reduction was variable among treatments. It decreased 1- to 4-fold, compared with the remaining viability after 5 d of storage. Moreover, the negative control conidia in 0.5% INEX-A lost 100% viability in both tested samples. Similarly, results showed high viability reduction among treatments, where additives were tested at the highest concentrations.

Comparing conidia viability after exposure with additives over time, significant differences among lactic acid at different concentrations were observed after 0 d (F_40_,_3_ = 3.899, *p* = 0.016), 5 d (F_32,3_ = 68.854, *p* < 0.001), and 30 d of storage (F_31,3_ = 34.112, *p* < 0.001). At time zero, 90% to 100% conidia viability was observed, where only control resulted in significantly (*p* = 0.05) higher viability, compared with that of 8 µg/mL lactic acid. After 30 d, 2 µg/mL was the concentration that retained the highest viability, followed by 4 µg/mL, whereas the control group and 8 µg/mL lactic acid showed the lowest viability (HDS, *p* = 0.05).

After exposure to acetone at different concentrations, conidia viability showed no differences among treatments after 0 d and 5 d (F_40,3_ = 0.867, *p* = 0.466 and F_38,3_ = 0.332, *p* = 0.802, respectively) but evidenced differences after 30 d of storage (F_29,3_ = 61.794, *p* < 0.001). Furthermore, the lowest acetone concentration (1%) retained the highest viability after 30 d of storage, followed by 5%, whereas acetone at 10% and control showed the lowest viability (HDS, *p* = 0.05).

Furthermore, after exposure to sugar at different concentrations, conidia viability showed no differences among treatments after 0 d (F_40,3_ = 1.925, *p* = 0.141). Differences were observed after 5 d (F_29,3_ = 34.802, *p* < 0.001) and 30 d of storage (F_30,3_ = 61.965, *p* < 0.001). Conidia exposed to the lowest sugar concentration (1%) and control retained the highest viability after 5 d of storage, followed by 5% sugar and control, whereas sugar at 10% showed the lowest viability (HDS, *p* = 0.05). After 30 d of storage, sugar at 1% retained the highest viability, followed by 5%, whereas sugar at 10% and control showed the lowest viability (HDS, *p* = 0.05).

### 3.2. B. bassiana Conidial Viability after Exposure to MGF

**Figure 1:**
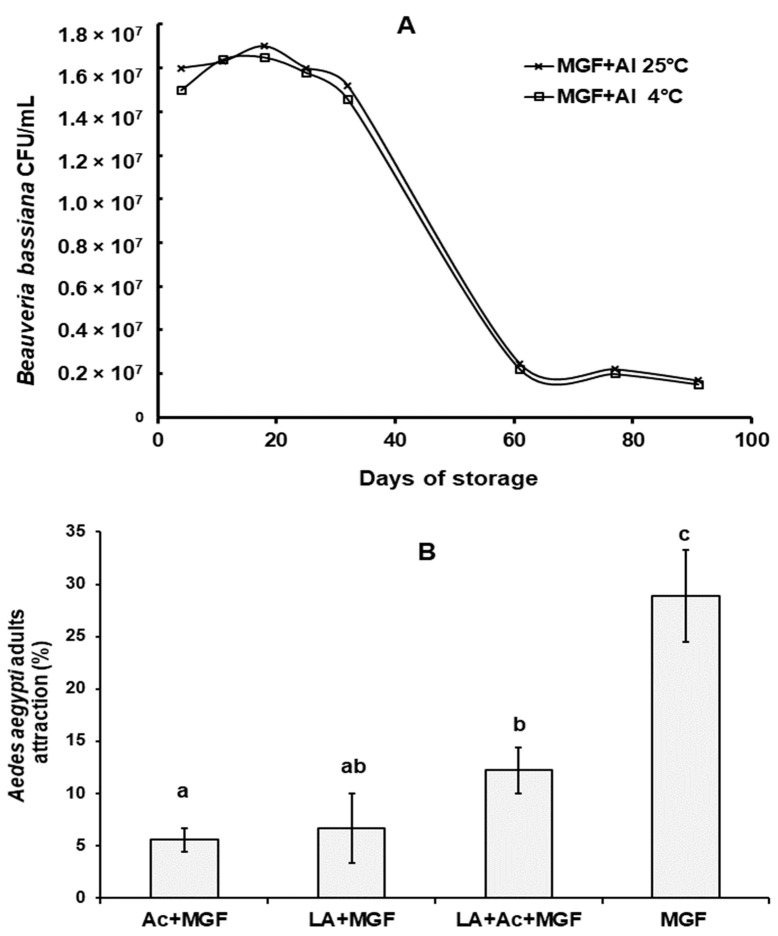
*Beauveria bassiana* BBPTG4 strain (**A**) conidia viability in microgranular formulation (MGF). Conidia viability was determined in MGF at time zero (4 d) (1.5 × 10^7^ conidia/mL as active ingredient (AI) with MGF) and up to 91 d storage at 25 °C ± 2 °C or 4 °C ± 1 °C, as explained in the text. (**B**) Percentages of *Ae. aegypti* adults attracted to different treatments. *Ae. aegypti* adults were exposed to MGF without additive, MGF + acetone (added at 1% (*w*/*v*) of MGF), MGF + 100 µL/g of lactic acid at 85%, and MGF + 100 µL/g of lactic acid at 85% + 1% acetone/g of MGF and attraction percentages determined, as explained in the text. Data represent mean + SEM of triplicate determinations from three independent experiments. Same letter on each column indicates that treatments are not significantly different (HSD Tukey test; *p* ˂ 0.05).

BBPTG4 conidia remained viable after mixing in MGF. On this formulation, conidia lost less than 10-fold viability (from 1.5 × 10^7^ conidia/mL to 1.6 × 10^6^ conidia/mL at 25 °C and from 1.7 × 10^7^ conidia/mL to 1.5 × 10^6^ conidia/mL at 4 °C) after 30 d of storage, regardless of the storage temperature (Figure 1A). After this, BBPTG4 conidia lost 15-fold (1.0 × 10^6^ conidia/mL) viability but remained stable up to 91 d of storage, regardless of the storage temperature. Untreated MGF, used as a negative control, did not develop microbial growth as contamination (data not shown).

### 3.3. Ae. aegypti Attraction Efficacy by Microganule-Formulated Additives

Among tested treatments, the highest *Ae. aegypti* attraction efficacy (*p* < 0.05) was observed after using MGF (Figure 1B). Treatment attractions were 18.9%, 23.3%, and 22.2% lower in MGF + acetone, MGF + lactic acid, and MGF + lactic acid + acetone treatments, respectively (Figure 1B). In addition, when conidia were combined with all MGF treatments but the negative control, their attractiveness was significantly (*p* < 0.05) higher than MGF + additives (Figure 1B).

### 3.4. Ae. aegypti Attraction and Mortality by MGFs with B. bassiana

**Figure 2:**
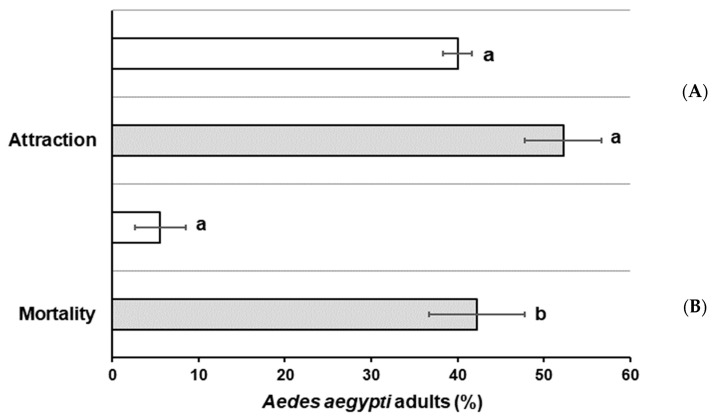
Percentage of *Ae. aegypti* adults attracted and killed by microgranular formulation (MGFs) with (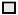 gray bar) or without (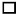 white bar) *Beauveria bassiana* as active ingredient (AI). (**A**) Attraction percentage and (**B**) mortality percentage after exposure to BBPTG4 on MGF. Data represent mean + SEM of triplicate determinations from three independent experiments. Same letter on each column indicates that treatments are not significantly different (Student *t* test; *p* ˂ 0.05).

After *B. bassiana* conidia (active ingredient) were mixed in MGF, named from now on as MGFs, for the sucrose use as additive, it was selected as the best treatment in Section 3.1. Results showed non-significant attraction (29.7%; *t*_(4)_ = 1.778, *p* = 0.150) compared with untreated MGFs (Figure 2A). Mortality of attracted mosquitoes by conidia was lower than 40% but significantly (*t*_(4)_ = 5.983, *p* = 0.04) higher than that of untreated MGFs (<5% mortality) (Figure 2B).

### 3.5. Ae. aegypti Attraction by MGFs with Spirulina *sp.*

**Figure 3:**
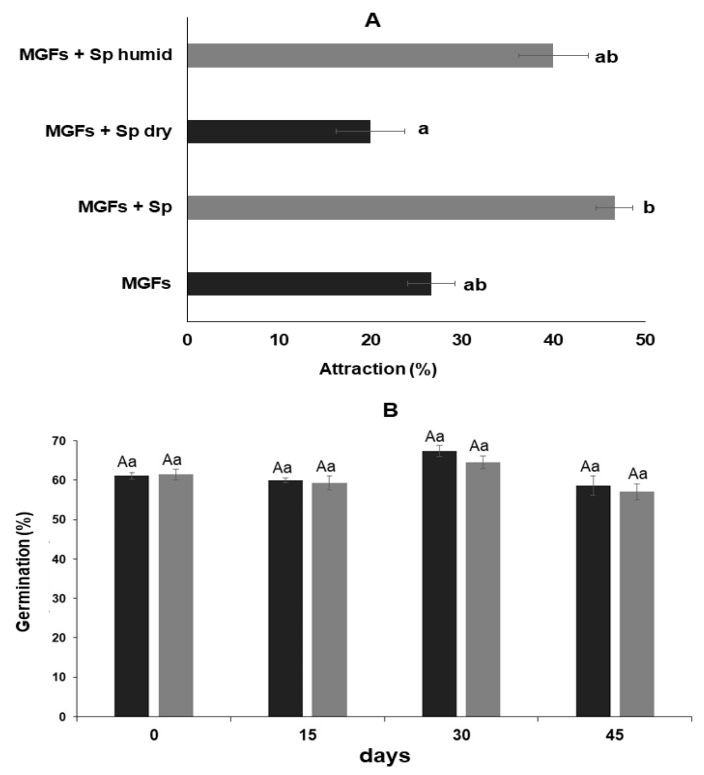
*Ae. aegypti* attraction by formulations. (**A**) Attraction percentage in *Ae. aegypti* adults of microgranular formulations plus *Spirulina*. Same letter on each column indicates that treatments were not significantly different (HSD Tukey test, *p* > 0.05). (**B**) *B. bassiana* conidia viability after different days of storage at different temperatures. Dark bars correspond to MGF + *Spirulina* at 25 °C ± 2 °C and gray bars represent MGF + *Spirulina* at 4 °C ± 2 °C storage temperature. Same letter on dark or gray column indicates not significantly different treatments (HSD Tukey test, *p* > 0.05). Data represent mean + SEM of triplicate determinations from three independent experiments. The same letter between columns in the same storage time indicates not significantly different treatments (Student *t* test; *p* ˂ 0.05). Treatments were MGF + *Spirulina* at 25 °C ± 2 °C and 4 °C ± 1 °C.

MGFs + Sp showed ~20% more attraction compared with MGFs (*p* > 0.05) and MGFs + Sp dry (*p* < 0.01) (Figure 3A). However, when testing dry or humid formulates, we did not detect significant differences between them (*p* > 0.05) (Figure 3A), although the humid formulate was 20% higher (Figure 3A).

### 3.6. B. bassiana Conidia Viability in MGFs with Spirulina *sp.*

*B. bassiana* conidia shelf life in MGF with sugar and *Spirulina* sp. (MGFs + Sp) was not reduced based on conidia germination percentage for up to 45 d (Figure 3B).

### 3.7. B. bassiana Conidia Viability in MGFs and Solid Formulations in Two Storage Conditions (25 °C and 4 °C)

Conidia viability means values on formulations after preparation (0 h or 24 h after preparation) at 4 °C were in the range of 2.9 × 10^7^ to 4.8 × 10^7^ CFU/g and 3.2 × 10^7^ to 4.8 × 10^7^ CFU/mL at room temperature, which were significantly (*p* < 0.01) lower, compared with the control (1.2 × 10^8^ CFU/mL). Conidia viability on MGF + sugar + *Spirulina* (4.5 × 10^7^ CFU/mL) and MGF + sugar (3.8 × 10^7^ CFU/mL) treatment data after storage at 4 °C did not show significant (*p* > 0.5) differences at time zero but they were different compared with all other treatments except solid formulation stored at 4 °C (4.8 × 10^7^ CFU/g).

**Figure 4:**
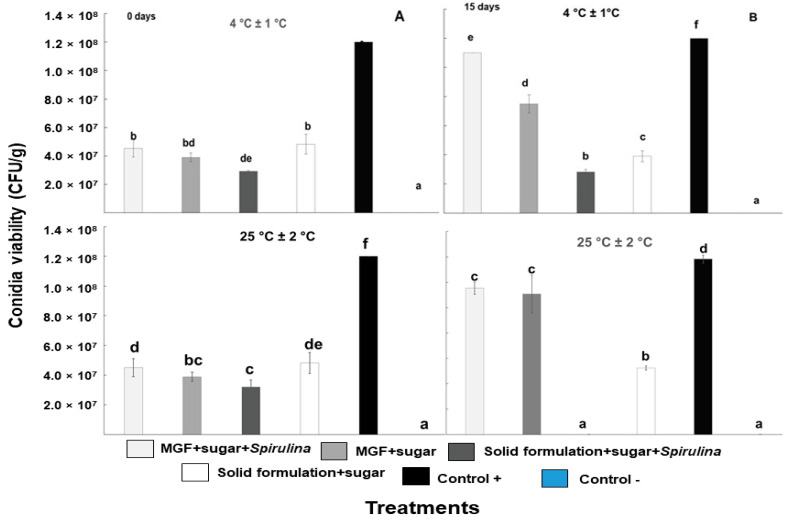

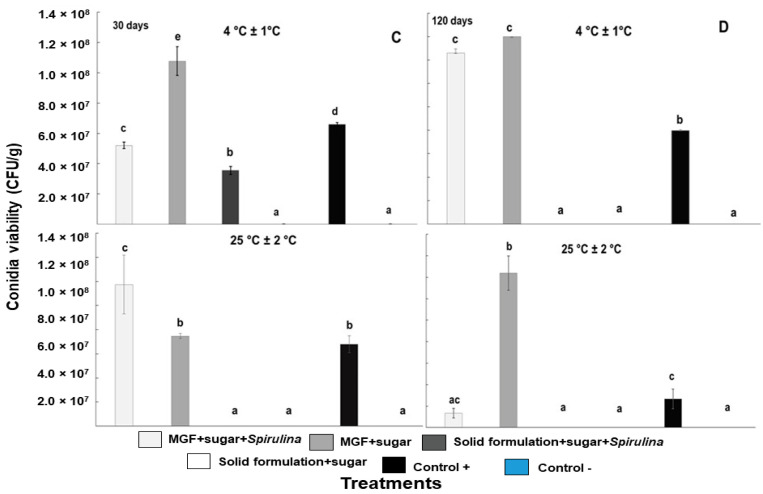
*Beauveria bassiana* conidia viability in microgranular formulation (MGF) at (**A**) 0 d, (**B**) 15 d, (**C**) 30 d, and (**D**) 120 d in colony forming units per gram (CFU/g). MGFs = MGF plus sugar, FS = solid formulation. Data represent mean + SEM of triplicate determinations from three independent experiments. Same letter between columns indicates that treatments are not significantly different (HSD Tukey test; *p* > 0.05).

Furthermore, solid formulation + *Spirulina* stored at 4 °C treatment showed lower viability (2.92 × 10^7^ CFU/mL) compared with all other treatments stored at the same temperature. Conidia viability at 25 °C in MGF + sugar + *Spirulina* (4.5 × 10^7^ CFU/mL) was slightly higher (*p* > 0.05) compared with solid formulation + sugar + *Spirulina* (3.8 × 10^7^ CFU/mL) or solid formulation + sugar (3.2 × 10^7^ CFU/mL) (*p* < 0.01). The positive control maintained its viability value (1.2 × 10^8^ CFU/mL), which was significantly (*p* < 0.05) higher than all other treatments (Figure 4A).

After 15 d of storage, conidia viability in both temperature treatments was significantly (*p* < 0.01) lower, compared with the positive control (1.2 × 10^7^ and 1.4 × 10^7^ CFU/mL, respectively). Conidia viability among formulations stored at 4 °C was significantly (*p* < 0.01) higher in MGF + sugar + *Spirulina* (1.1 × 10^8^ CFU/mL) compared with MGF + sugar, solid formulation + sugar + *Spirulina*, and solid formulation + sugar treatments (7.5 × 10^7^, 2.8 × 10^7^, and 3.9 × 10^7^ CFU/mL, respectively).

Regarding formulations stored at 25 °C, MGF + sugar + *Spirulina* and MGF + sugar showed significantly (*p* < 0.01) higher viability (1.1 × 10^8^ and 1.11 × 10^8^ CFU/mL, respectively), compared with solid formulation + sugar (5.2 × 10^7^ CFU/mL) and solid formulation + sugar + *Spirulina* treatments, where viable conidia were not detected (0 CFU/mL). Conidia viability in the positive control was significantly (*p* < 0.01) higher, compared with all other treatments (1.4 × 10^8^ CFU/mL) (Figure 4B).

Results obtained after 30 d of storage at 4 °C or 25 °C, showed lower conidia viability among most formulations. However, MGF + sugar + *Spirulina* treatment had higher viability after storage at 25 °C temperature (1.17 × 10^8^ CFU/mL). Similarly, MGF + sugar at 4 °C and MGF + sugar + *Spirulina* at 25 °C (1.1 × 10^8^ and 1.2 × 10^8^ CFU/mL, respectively), resulted in higher conidia viability compared with other treatments. In contrast, we did not observe viable conidia in solid formulation + sugar treatment stored at 4 °C temperature and solid formulation + sugar + *Spirulina* and solid formulation + sugar stored at 25 °C (Figure 4C).

After 120 d of storage, the highest conidia viability was detected in MGF + sugar and MGF + sugar + *Spirulina* (1.6 × 10^8^ and 1.5 × 10^8^ CFU/mL, respectively; *p* > 0.5) treatments at 4 °C, compared with all other treatments (*p* < 0.01). Conidia in the positive control maintained their viability (8.0 × 10^7^ CFU/mL) and was significantly (*p* < 0.01) higher compared with all other treatments (Figure 4D). MGF + sugar + *Spirulina* and MGF + sugar (6.7 × 10^5^ CFU/mL and 7.2 × 10^6^ CFU/mL, respectively) formulations stored at 25 °C showed high conidia viability but lower compared with those stored at 4 °C temperatures. Furthermore, MGF + sugar + *Spirulina* stored at 25 °C, showed significantly (*p* < 0.01) higher viability compared with all other treatments, but it was not different compared with the positive control (1.33 × 10^6^ CFU/mL) (Figure 4D).

### 3.8. Aedes aegypti Biocontrol by MGF and FS with B. bassiana Conidia

**Figure 5:**
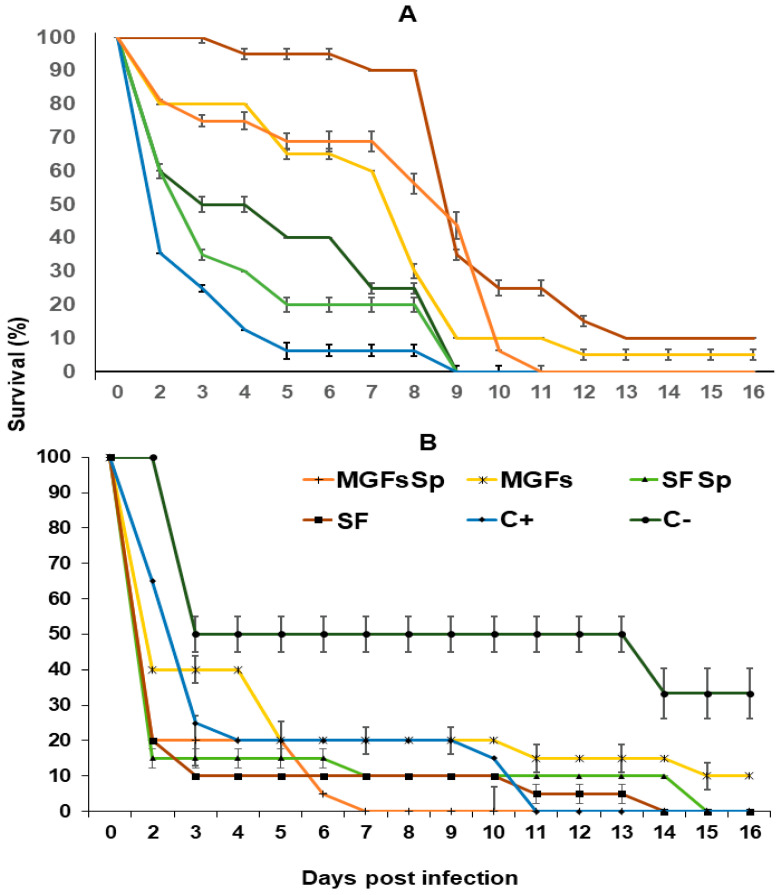
Survival percentage of *Aedes aegypti* adults exposed to four different formulations of the *B. bassiana* BBPTG4 strain stored for (**A**) zero days and (**B**) 30 d. The four different formulations stored at 4 °C and 25 °C. Data represent mean + SEM of triplicate determinations from three independent experiments. MGF + Sp = microgranular formulation + *Spirulina*, MGFs = plus sugar, SF = solid formulation, C+ = positive control, C− = negative control.

We evaluated the insect survival of four formulations against *Ae. aegypti* adults. After 5 d of exposure, the positive control (*B. bassiana* suspension at 1 × 10^8^ conidia/mL) showed a survival percentage of 6.25%, whereas MGF + sugar + *Spirulina* and solid formulation + sugar + *Spirulina* resulted in 40% and 20% survival, respectively. High survival was detected by solid formulation MGF + sugar + *Spirulina* and solid formulation MGF + sugar treatments, showing above 65%. After 9 d of exposure, insect survival was reduced in treatments without *Spirulina*; MGF + sugar and solid formulation + sugar showed 5% and 10% survival after 16 d, respectively (Figure 5A).

After 30 d of storage, conidia treatments also affected mosquitoes. In this regard, MGF + sugar + *Spirulina* was the most effective, since after 7 d of exposure none exposed adult survived, whereas solid formulation + *Spirulina* and solid formulation showed 10% adult survival and MGFs and positive control resulted in 20% adult survival. The positive control, solid formulation + sugar, and solid formulation MGF + sugar + *Spirulina* treatments reduced the adults’ survival to zero after 11 d, 14 d, and 15 d of exposure, respectively, unlike the negative control, which after day 16 maintained 33.3% survival (Figure 5B).

## 4. Discussion

For a successful vector control application, we aim to improve entomopathogenic fungi shelf-life storage, increase insect attraction, and maintain viability in open places [19]. In the present study, we developed an effective “attraction–infection–kill” formulation against *Ae. aegypti* by combining *B. bassiana* conidia, attractants, and a surfactant. We evaluated the active ingredient´s stability during storage at room temperature in solution and in MGF. We first assessed the cytotoxicity of the previously reported attractants to *Ae. aegypti* acetone, lactic acid, and sugar against *B. bassiana* conidia [23]. Results showed the absence of cytotoxic effect, and conidia remained in suspension with the attractant and the surfactant (0.5% INEX-A). After 5 d of storage at 25 °C ± 2 °C, conidia significantly lost their viability (from 4.3 × 10^8^ to 0.29 × 10^5^ conidia/mL), regardless of the treatment.

This effect may be the result of conidia permeability changes, since they were stored in suspension at room temperature, allowing enzyme (proteases, peptidases, chitinases, lipases, and phospholipases) and other metabolite production [42]. After 30 d of storage, conidia in both controls (mixed in 0.5% INEX-A) lost 100% viability. This may be the effect of the surfactant by favoring conidia germination and eventually, their death due to oxygen and nutrients scarcity [43], since after 5 d of storage ~6.0 × 10^5^ conidia/mL remained viable. This adverse effect of attractants on conidia viability increases the need for formulations and attractants that do not alter it.

Regardless of the attractant, conidia significantly lost viability in a dose–response manner, mainly in the lactic acid and sugar treatments. Osmotic stress may have played an important role in conidia over the time, probably due to super-saturation and cell wall turgor lost [44]. However, the highest *B. bassiana* conidia viability after 30 d was observed in treatments with 1% and 5% acetone, plus 1% sugar.

In general, conidia survival is reduced in many conventional formulations [18]. Results of the present study demonstrated that MGF provided stability to *B. bassiana* conidia after storage at room temperature, where 80% viability remained after 32 d of storage. Moreover, MGF enhanced conidia viability and mosquito attraction. Furthermore, viability reduction was observed regardless of the storage temperature, demonstrating that temperature was not a determining factor of conidia stability.

According to our results with sugar in MGF, this formulation increased *Ae. aegypti* adult attraction, which may result in one commercial formulation for mosquito control in domestic traps with some modifications. Since these traps have been used for *Ae. aegypti* females to lay eggs, and acetone has shown up to 78% attraction to females despite its volatility [23], it may be important to evaluate its efficacy versus application over time, when conidia remain viable in the trap and CO_2_-bioproducing system (BG CO_2_ Mosquito Trap | Nixalite accessed on 30 June 2022).

The most important aspect for any formulation based on a biocontrol agent is the product efficacy. Several studies reported that unformulated *B. bassiana* conidia suspensions at 1 × 10^8^ and 6 × 10^8^ conidia/mL, directly applied on a filter paper against *Ae. aegypti* adults, caused 89% to 90% mortality, where surviving infected adults increased the biocontrol rate by co-infection via copula [8]. Indeed, they reported *Ae. aegypti* adult mortality to up to 40%, after testing in a 123.8 cm^3^ infection system [8]. In our laboratory, a domestic trap with MGF at 1 × 10^8^ conidia/g, placed in an area almost 10 times higher (1205.1 cm^3^), was used, reaching a similar mortality (42%). Therefore, the function of the formulation was fulfilled, not only due to the stability of the formulation but also to the possibility of increasing the doses generally used.

In fact, when a lower concentration (2.4 × 10^4^ conidia/g) was used in the same infection system, using MGF as a base, the attraction was not significantly increased by 20%. When evaluating the MGF humid formulation, we found that it improves the attraction percentage by 20%, which may be due to the reactivation of the microorganisms (*Spirulina* and others). However, the difference was not significant compared with MGF + *Spirulina* dry. Although granule wetting did not enhance the attraction (although a trend was observed), this is consistent with a series of products on the market, whose application depends on wetting for the reactivation of the control agent and the volatilization of attractants.

Our results showed that the addition of a low concentration of *Spirulina* sp. (3.8%) enhanced the effectiveness of *B. bassiana* for *Ae. aegypti* biocontrol. Others have also shown that the addition of phage-stimulating additives, such as chitin in the formulation with *B. thuringiensis*, reversed the toxicity of the pathogen, perforating peritrophic membranes of the midgut in larvae and increasing the accessibility of toxins to epithelial cells [45]. However, in our study, we used inexpensive attractants such as sugar or *Spirulina* sp., which showed promising results.

Research on insect control, particularly culicids, focuses on effectivity. For instance, a pet trap covered with fabric impregnated by a synthetic bait (AtrAedes, Agrisense Ltd., Cardiff, UK) in combination with *Metarhizium* *anisopliae* (Metsh^®^) against *Ae. aegypti* females, under a controlled environment (intra-domicile conditions), reported up to 68% mortality [27,29]. Another reported trap system using black plastic flower pots (which have larvicidal and adulticidal activities, included a juvenile hormone analogue (pyriproxyfen) to attract gravid *Ae. aegypti* females. Experiments using this system included combinations with *B. bassiana* spores, and were performed under three blunt screens, resulting in lower *Ae. aegypti* adult survival percentages [46]. Nevertheless, the conidia shelf life on this fabric-based *Ae. aegypti* bait was rather low. Results showed that conidia viability was preserved for 45 d at 25 °C.

To develop a formulation that provides *B. bassiana* conidia stability, viability, and effectiveness, we tested one microgranular (MGF) and one solid formulation, which contained vegetable fat, *Spirulina* sp., or coconut fiber. One study reported a *M. anisopliae* formulation to control *Rhipicephalus microplus* (Canestrini) (Acari: Ixodidae). It contained 10% oil to produce a homogeneous emulsion and field dispersion, providing adhesion at the application time [47]. We also developed emulsions with *B. bassiana* conidia and after their exposure to *Ae. aegypti* adults we observed repellency, due to the viscosity it presented (data not shown). This demonstrated that, in addition to being careful about the technology for the preparation and ingredients used, we must consider the insect´s ethology, when using formulations.

In addition, the use of coconut fiber, as the one used in our work, in formulations to improve *Pseudomonas* *chlororaphis* shelf life, maintained bacteria viability for up to 8 months [48]. In contrast, in the present study, we observed that solid formulation significantly decreased *B. bassiana* conidia viability from 15 d of storage and at 30 d we did not find viable conidia (*p* < 0.01). This loss of viability may suggest that this type of ground substrate does not generate a homogeneous mixture, allowing conidia exposure to abiotic factors (light, increased temperatures, and low humidity).

The most effective formulations in our study were MGF + sugar + *Spirulina* and MGF + sugar, at 4 °C which contained flour, corn starch, and vegetable oil. In these formulations, *B. bassiana* conidia viability was maintained at 1.4 × 10^8^ CFU/mL and 1.6 × 10^8^ CFU/mL after 120 d of storage. Viability increased probably because of *Spirulina*, which may have provided stability to conidia due to their fat content (11% of lipids) [20]. We also found the viability of 7.20 × 10^6^ CFU/mL for MGF + sugar at 25 °C (an adequate concentration to reach mortalities greater than 40%), which was statistically different from the rest of the treatments stored at 25 °C. This may be due to different additives that provided protection, unlike solid formulation. In conclusion, the addition of *Spirulina* increases the attraction of *Ae. aegypti*, which correlated with its mortality. However, it does not provide any conservative benefit to the MGF, as it could be seen in this experiment with the treatments MGF + sugar + *Spirulina* at both temperatures and MGF + sugar at 4 °C.

MGF + sugar + *Spirulina* and MGF + sugar treatments showed values of 4.5 × 10^7^, 1.10 × 10^7^, 5.20 × 10^7^, and 1.46 × 10^8^ CFU/mL and 3.89 × 10^7^, 7.49 × 10^7^, 1.08 × 10^8^, and 1.60 × 10^8^ CFU/mL at 0 d, 15 d, 30 d, and 120 d, respectively, at 4 °C. The same trend was observed in samples stored at 25 °C for in the same treatments. It was evident that the best MGF formulations were those stored at 4 °C with conidia at a range of 1 × 10^8^ CFU/mL after 120 d, whereas for the granular formulation treatments stored at 25 °C, the final viability values in MGF + sugar + *Spirulina* and MGF + sugar treatments at 25 °C were 6.67 × 10^5^ CFU/mL and 7.20 × 10^6^ CFU/mL, respectively, after 120 d of storage. After comparing these results with conidia viability in the positive controls, in general they showed decreasing and lower viability values. This confirms that when powdered vegetable oil was added to the formulations, conidia viability was higher after storage. In this regard, it has been emphasized that bioinsecticide storage induces a slow metabolism, especially when they are stored under refrigeration [48]. This agrees with our MGF results, which suggested a slower conidia metabolism but not dormancy, as observed among other microorganisms under −20 °C storage. This also suggested that the addition of powdered vegetable oil to our formulations reduced conidia loss of moisture when they are stored in microgranules. However, due to the physical conidia hydrophobicity, conidia in an oil-based emulsion may be kept viable for longer storage periods [49,50]. In this regard, *Cordyceps fumosorosea* (Wize) kept germination and viability after storage, which was associated with the addition of corn oil to the formulation [51]. Other authors have suggested that the adequate amount of oil for *B. bassiana* conidia formulations should be from 1% to 2% [52]. Nevertheless, the amount tested in our granular formulation was higher. This may be important since keeping any pesticide under freezing conditions is more expensive than at 4 °C. Our results suggest that formulations with ingredients such as powdered vegetable oil may help to improve *B. bassiana* conidia shelf life.

Regarding formulations effectiveness, it was observed that at time 0, the positive control was effective in terms of the survival percentage of *Ae. aegypti*, probably due to an increased conidia exposure to mosquitoes than in formulation. We also observed that the most effective treatments to decrease survival at time 0, were those formulated with *Spirulina* (MGF + sugar + *Spirulina* and solid formulation + sugar + *Spirulina*). However, the best treatment after 30 d was MGF + sugar + *Spirulina*, since at 7 d after exposure, it caused a mortality of 100%, unlike the other treatments, which agrees with the viability found in these treatments in the range of 2 × 10^7^ to 1 × 10^8^ CFU/mL. Other authors reported that the use of *B. bassiana* conidia in a liquid formulation with oil, reduced *Ae. aegypti* survival by 61% to 69%, after 10 d of exposure in a semi-field system with small cages [52]. Furthermore, after using black fabrics impregnated with *B. bassiana* conidia in PET plastic traps, an increase in *Ae. aegypti* attractiveness from 31% to 66% and a 52% decrease in survival was observed after 120 h [27].

An important aspect is to demonstrate that the system works after storage for a long time, by validating the effectiveness in the field, as well as identifying if the additives used in our study provide residual activity in the field. Previous reports have demonstrated that *B. thuringiensis* microencapsulation, developed for *Choristoneura rosaceana* (Harris) larvae control, caused up to 33% mortality immediately after application, which persisted for up to 14 d, where activity was affected due to biotic factors (precipitation, temperature, and solar radiation) [53]. These results confirm the importance of identifying the proper time and place of application. In the case of MGF, it appears that these types of formulations may have to be tested outside houses, under trees or bushes and perhaps garages with dark places, places for which flies have an affinity.

We consider that although a reduction in conidia viability in formulations developed in this study was observed, this commonly produces unfavorable results. However, it is acceptable for use in mosquito control and has appropriate characteristics to continue with the investigation. For example, its high attraction and mortality are characteristics that make this *B. bassiana*-based bioformulation, an alternative to reduce the use of chemical agents and increase public awareness about the benefits of the use of biological products, whose action does not compromise human health. In fact, according to the effectiveness data of previously mentioned species (*C. fumosorosea*, *B. bassiana,* and *B. thuringiensis*), this bioinsecticide still possesses functional viability.

The use of a formulation that attracts mosquitos in a trap, combined with conidia, may increase the target infection rate. Since a tested trap is easy to make and install, its use in the home or field seems feasible. There are many traps that catch mosquitos but the idea to attract and expose them to *B. bassiana* conidia is to increase the infection/mortality rate, knowing transmission feasibility via copula [8]. After testing an attractant device with *M. anisopliae* for the *Ceratitis capitata* (Wiedemann) biocontrol under field conditions, results showed a fruit fly population reduction and that the inoculation dishes needed mid-season replacing to provide protection for the entire season [54]. We believe that similar replacement over the time for the trap-fungi formulation described in this study may help to increase mosquito infection and their biocontrol.

## 5. Conclusions

In conclusion, the combination of formulated granules with *B. bassiana* as an active ingredient attracted and infected *Ae. aegypti* adults in a domestic trap, where conidia remained viable for up to one month at 25 °C ± 2 °C. Our results open a new strategy to develop a formulation as a vector control management tool. Formulation improvements in the manufacturing process generates knowledge on the ingredient’s effective combination for insect target attraction, mortality, and *B. bassiana* conidia viability, as well as the preparation and equipment use. Taken together, our results provide the elements to obtain a commercial product for *Ae. aegypti* biocontrol.

## Figures and Tables

**Table 1:** Ingredients for mixing *Beauveria bassiana* conidia on microgranular formulations.

Ingredients	MGF	MGF + Sp	SF	SF + Sp
*B. bassiana* (AI; 1 × 10^8^ conidia/g)	0.9 g	0.9 g	0.9 g	0.9 g
Nixtamalized corn flour	21.5 g	8.14 g	-	-
Cornstarch	21.5 g	8.14 g	-	-
Purified water	30 mL	30 mL	-	-
Corn oil	16 mL	16 mL	-	-
Sucrose (1%)	-	0.9	0.9 g	0.9 g
Vegetable grease (25%)	-	22.5 g	22.5 g	22.5 g
*Spirulina* (3.8%)	-	3.4 g	-	3.4 g
Coconut fiber	-	-	62.3 g	58.9 g
Total	90 g			

MGF = microgranular formulation, MGF + Sp = MGF with *Spirulina*, SF = solid formulation, SF + Sp = solid formulation with *Spirulina*, AI = *B. bassiana* conidia as active ingredient.

**Table 2:** *Beauveria bassiana* conidia shelf-life on microgranular formulations after storage at 25 °C and 4 °C.

Treatments	Treatment Codes and Storage Temperatures 25 °C ± 2 °C and 4 °C ± 1 °C
MGF + AI + sugar + *Spirulina*	MGF + sugar + *Spirulina*
MGF + AI + sugar	MGF + sugar
FS + AI + sugar + *Spirulina* + coconut fiber	Solid formulation + sugar + *Spirulina*
FS + AI + sugar + coconut fiber	Solid formulation + sugar
Positive control (0.5% INEX-A + AI)	Positive control
Negative control (0.5% INEX-A)	Negative control

MGF = microgranular formulation, AI = *B. bassiana* conidia as active ingredient, see Table 1 for conidia per gram in the final formulation.
